# The Production and Evaluation of an Electrochemical Sensors for Strychnine and Its Main Metabolite Strychnine N-Oxide for Their Use in Biological Samples

**DOI:** 10.3390/molecules27061826

**Published:** 2022-03-11

**Authors:** Bakhtiyar Qader, Issam Hussain, Mark Baron, Rafael Estevez-Brito, John Paul Cassella, Jose Gonzalez-Rodriguez

**Affiliations:** 1 Sulaimani Medicolegal Institute, Qanat Street, Kurdistan Regional Government, Sulaimani, Sulaymaniyah 46001, Iraq; bakhtyar88@gmail.com; 2 School of Chemistry, Joseph Banks Laboratories, University of Lincoln, Lincoln LN6 7DL, UK; mbaron@lincoln.ac.uk; 3 School of Life Sciences, Joseph Banks Laboratories, University of Lincoln, Lincoln LN6 7DL, UK; ihussain@lincoln.ac.uk; 4 Department of Physical Chemistry and Applied Thermodynamics, University of Cordoba, 14014 Córdoba, Spain; rafa.eb@outlook.es; 5 Department of Life Sciences, School of Science, Institute of Technology Sligo, F91 YW50 Sligo, Ireland; cassella.john@itsligo.ie

**Keywords:** strychnine, strychnine N-Oxide, in vitro analysis, forensic analysis, electrochemistry

## Abstract

Strychnine (STN) and its major metabolite Strychnine N-Oxide (SNO) were examined electrochemically. Both parent compounds and its major metabolite showed electroactivity on glassy carbon electrodes using CV and DPV techniques. One oxidation peak at 1008 mV was observed for STN with the optimum peak intensity at pH 7. SNO produced two oxidation peaks, at 617 mV and 797 mV, at pH 5. The peaks demonstrated irreversible behaviour and the irreversibility of the system was confirmed at different scan rates. A calibration curve was produced for both CV and DPV measurements and the sensitivity of the proposed EC method was good compared with previous electrochemical and non-electrochemical methods. The precision of oxidation peak of STN using the STN-MIP method produced a maximum value of 11.5% and 2.32% for inter-day and intraday %RSD, respectively. The average% recovery was around 92%. The electrochemical method has been successfully applied to the determination of STN in spiked plasma and urine samples. For SNO, both anodic peaks of SNO demonstrated irreversible behaviour. A different sweep rate was used for calculating the number of ‘transfer electrons’ in the system; based on this, the mechanism of oxidation reaction was proposed. Calibration curves for both oxidative peaks were produced using DPV measurements. The second anodic peak demonstrated high linearity and precision with %RSD < 1.96%.

## 1. Introduction

Strychnine (STN) is a bulk alkaloid derived from the seeds of the tree Strychnos nux-vomica which mainly grows in tropical regions as in India and Southern Asia. It is a white, odourless, bitter-tasting crystalline powder. STN dissolves well in acidic solutions while its dissolution in either water or ether is weak [1,2]. Strychnine is still used as a rodenticide and pesticide to kill birds in some countries, but it has been banned in most of developed countries [3]. In some prescription of traditional Chinese medicine, STN has been effectively used in treating some types of nerve diseases, joint pain, rheumatic diseases, allergic symptoms and inflammations. In addition, STN has a little therapeutic significance against some tumours [1,2,4,5]. However, the clinical use of STN has been limited due to its high toxicity. It is reported to be fatal in a dose of 30–120 mg and death usually happens by respiratory failure [1]. Severity of STN poisoning can be assessed from STN concentration in blood; it could be toxic if the blood concentration is less than 2 mg/L (6 µM) and become life threatening if the concentrations rise to 2 and 10 mg/L.

A study conducted upon zebra fish embryos, reported that strychnine N-oxide (SNO) has a lesser toxic effect than strychnine; therefore, SNO is used extensively in clinical practice [6] since its curative use is still effective [7]. SNO is a derivative as well as the major metabolite of strychnine.

Ingestion is a common route of STN toxicity followed by poisoning through inhalation and skin absorption. Strychnine is rapidly absorbed in the stomach and small intestine and then extensively metabolized by the liver into many metabolites. Nearly 20% of the circulating STN is excreted by kidneys within 24 h and is unchanged [1]. 

Strychnine is absorbed in the stomach and small intestine after ingestion quite rapidly [8]. Then, STN leaves the bloodstream immediately and is quickly distributed to the tissues by binding to plasma proteins. A peak plasma concentration is usually found 2–4 h after ingestion. STN half-life in plasma is 15.9 h. Excretion starts a few minutes after being introduced in the body. Finally, STN is excreted from the body completely after 48 to 72 h [9].

As soon as STN reaches the liver, it is extensively metabolized and detoxified by the liver microsomal enzyme system (cytochrome P-450 2B) in the presence of NADPH and Oxygen to form several metabolites [10,11,12]. In vitro metabolic studies using rat liver microsomes revealed 22 different metabolites of strychnine, mainly including N-oxidation of STN as strychnine-N-oxide (SNO), strychnine-2-epoxide-N-oxide, 2-OH-strychnine-N-oxide, 3-OH-strychnine-N-oxide, and hydroxyl-strychnine-N-oxide [12]. Another study confirmed that the major metabolite in the STN pathway is SNO and accounted for approximately 15% of the metabolized STN [1].

Key analytical methods for the analysis of STN and its major metabolites in pharmacological and toxicological studies include nuclear magnetic resonance (1H-NMR) [13], thin layer chromatography (TLC) [14,15,16,17], high performance liquid chromatography (HPLC) [11,18,19,20,21,22], liquid chromatography–mass spectrometry (LC-MS) [8,10,23,24,25], gas chromatography–mass spectrometry (GC-MS) [26,27,28] and capillary electrophoresis (CE) [5,29] have been reported.

The voltammetric behaviour of STN has been studied with a potentiostat-galvanostat using a pyrolytic graphite electrode, saturated calomel electrode as a reference and a platinum electrode as an auxiliary electrode. Typical redox peaks were obtained at −0.371 V and −0.406 V in a 0.05 M Gly-HCl buffer solution at pH 3.0 using a scan rate of 100 mV s^−1^ and a pre-accumulation time of 5 min. A linear regression line was obtained for the concentration range 0.334–36.74 µg mL^−1^ with a LOD of 0.003 µg mL^−1^ and %RSD = 2.3% precision. This method has also been applied to the determination of STN for *Strychno nux-vomica* seed extracts with a recovery rate of 99.8% [30]. Various materials and methods have been studied for the fabrication and modification of working electrodes in electrochemistry to magnify the peak intensity and to reduce LOD [31]. This is the reason behind the modification of a carbon paste electrode with gold nanoparticles (GNPs/CPE) to determine STN in Strychnos nux-vomica seeds. CV, DPV, chronocoulometric techniques were used for measuring STN in a 0.2 M B-R buffer at pH 7.0 achieving a LOD of 0.15 µg mL^−1^ by DPV [31]. Another reported method was used to separate and determine low levels of STN and brucine in rat serum. The system composed of a poly(dimethysiloxane) (PDMS) fibre dynamically modified by Brij35 on a carbon fibre microcolumn as working electrode was inserted into the microchip.

Following liquid–liquid extraction of the analytes from rat serum, the sample was measured with CV in 70 mmol L^−1^ acetate buffer (pH 5.5) containing 0.01% (*v*/*v*) Brij35. An anodic peak was detected at 1.17 V for STN with a Limit of detection 1 µM and a mean recovery of 87.4% [32].

To the best of our knowledge, no electrochemical method has been reported to date for the analysis of its major metabolite, SNO. Moreover, no molecularly imprinted polymer-based electrochemical detection has been reported for neither STN nor its metabolites in the literature.

## 2. Results and Discussion

### 2.1. Electrochemical Behaviour of Strychnine

Prior to the preparation of the strychnine MIP (STN-MIP), the electrochemical behaviour of strychnine was investigated by CV and DPV using oxidation scans at the bare GC electrode. Clearly defined oxidation peaks were recorded for STN indicating that they were electroactive on a GC electrode [30,31,32]. Figure S1A,B in Supplementary Materials, show typical voltammogram peaks for 1mM STN in Britton-Robinson (B-R) buffer solution at pH = 7.

The influence of pH on the oxidation peaks of STN on a GC electrode was also examined in a pH range of 3–10 using B-R (Figure S2). It was determined that the pH plays a role in the electrochemical behaviour of STN. The potential peak of STN shifted toward the negative direction with the increasing pH and the peak current intensity also changed. The relationship between pH value and peak potential was linear (Figure S3A), indicating that the same number of electrons and protons are involved in the overall electrode reactions [31,33]. The optimal pH value (Figure S3B) was found to be 7.0 for the STN, showing the highest peak intensity. This observation follows exactly the behaviour reported by Behpour et al. [31] The peak potential at this value was 1008 mV.

The effect of scan rates (ʋ) on the oxidation peak of STN was studied within the range 10–1000 mV s^−1^ (Figure S4).

The peak potential was shifted positively when the scan rate was increased, confirming the irreversibility of the oxidation reaction.

The logarithm of peak intensity (log I_p_) was in a linear relationship with a value of logarithm of scan rate (log ʋ), as shown in Figure S5.

**Figure 1:**
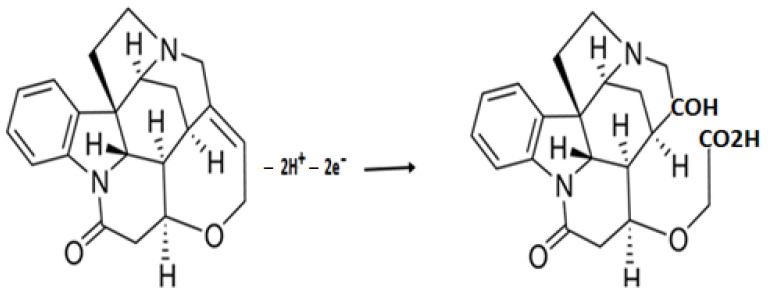
Suggested oxidation mechanism of strychnine at a glassy carbon electrode.

The calculations to assess the number of transferred electrons in the oxidation reaction can be found in the Supplementary Materials. The number of electrons participating in the oxidation of strychnine was identified as 2, which was consistent with the values previously identified in the literature. The suggested mechanism for the oxidation of strychnine is shown in Figure 1.

A full analytical validation of the direct electrochemical oxidation of strychnine can be found under Supplementary Materials and it will be used at a later stage to assess the advantages of the STN-MIP sensor. Figures S6–S9 and Tables S1–S4 in Supplementary Materials show the results obtained for this validation.

### 2.2. Electrochemical Behaviour of Strychnine N-Oxide Strychnine N-Oxide (SNO) Voltammetry

The voltammetric behavior of the SNO was studied using a bare GC electrode by both CV and DPV in B-R buffer solution. The influence of pH on the oxidation peaks of SNO was investigated in 0.1 M B-R buffer in a pH range of 3–8 using DPV (Figure S10). The peak potential shifted in a negative direction, with a decreasing pH value. A plot of peak potential vs pH (Figure S11) confirmed that both peaks linearly changed with the increasing pH value from 3 to 8, obeying the following equations:

Ep1 = −0.0592 pH + 0.8545,       r^2^ = 0.983 (1)

Ep2 = −0.044 pH + 0.9616,       r^2^ = 0.998 (2)

The linearity changes between peak potential and pH confirmed that protonation participated in the overall reaction occurring at electrode surface [30,33,34,35].

Moreover, the current response for the first oxidation peak increased with acidity of the buffered media levelling-off at pH values of 4. Meanwhile, the current response in the second oxidation peak reached a maximum intensity at pH 5. Therefore, pH 5 was used for rest of experimental study (Figure S12). The peak potentials at this pH were 617 mV and 797 mV.

To elucidate the mechanism of reaction, the influence of scan rate (ʋ) on the SNO oxidation peaks was studied within the range 50–1000 mV/s using CV measurements (Figure S13). The anodic peaks were shifted positively when the scan rate was increased, confirming the irreversibility of the oxidation reactions.

To assess the type of reaction happening at the electrode surface and to evaluate whether they were adsorption- or diffusion-controlled, a plot of square root of scan rate and peak current intensity (I_p_) was performed. This demonstrated a liner relationship between scan rate and current response, suggesting that the reaction is diffusion-controlled. Additionally, the values of logarithm of the first anodic peak intensity (log I_p_) vs the logarithm of the scan rate offered a linear relationship with a value logarithm of the scan rate (log ʋ), as shown in Figure S14; the linearity expressed in the equation was as follows:

Log I_p_ = 0.7101 log ʋ + 1.1502,       r^2^ = 0.9959 (3)

This indicated that the slope of the equation, 0.71, is located between the theoretical value of 0.5 (diffusion control) and the value of 1 (adsorption control). This observation is suggesting that the reaction process at the electrode surface is a mixed adsorption–diffusion controlled mechanism [34,36,37].

As shown previously, the calculations to assess the number of transferred electrons in the oxidation reaction can be found in the Supplementary Materials. The number of electrons participating in the oxidation of strychnine is approximately 2.

**Figure 2:**
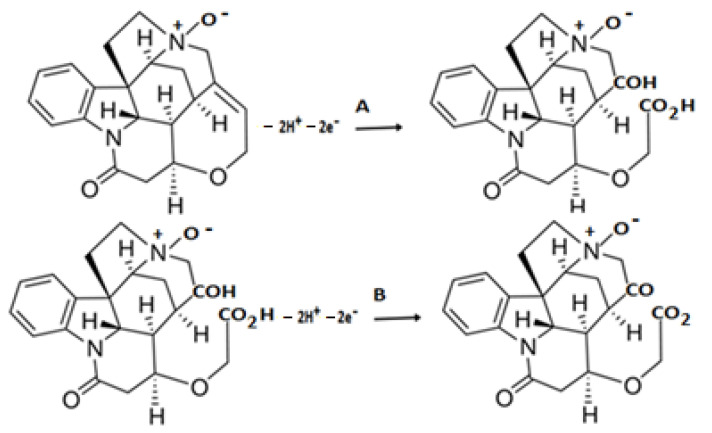
Suggested mechanism of SNO oxidation at GC electrode; (**A**) for the first oxidation peak and (**B**) for the second oxidation peak.

The mechanism of oxidation of SNO on the GC electrode surface is proposed in Figure 2.

A full analytical validation of the direct electrochemical oxidation of strychnine N-Oxide can be found under Supplementary Materials and will be used at a later stage to assess the advantages of the STN-MIP sensor (Figures S15–S17 and Table S5).

### 2.3. Strychnine Molecularly Imprinted Sensor (STN-MIP Sensor)

#### 2.3.1. Selection of Functional Monomers

Conducting polymers (CPs) are more commonly used for the design of MIP due to their optical, electronic, magnetic and electrical characteristic properties and also for their excellent ability to shift between undoped (insulating reduced) and doped (conducting oxidized) [38]. Among CPs, polypyrrole (PPy) has been most frequently utilised as the monomer for MIP sensors since PPy electric conductivity is good and can successfully entrap a broad range of templates owing to its electrochemical redox activity, even in pH-neutral solutions [37]. Hence, Pyrrole (Py) was chosen as a monomer with a strychnine template for MIP design.

#### 2.3.2. Fabrication of the STN Imprinted Sensor

**Figure 3:**
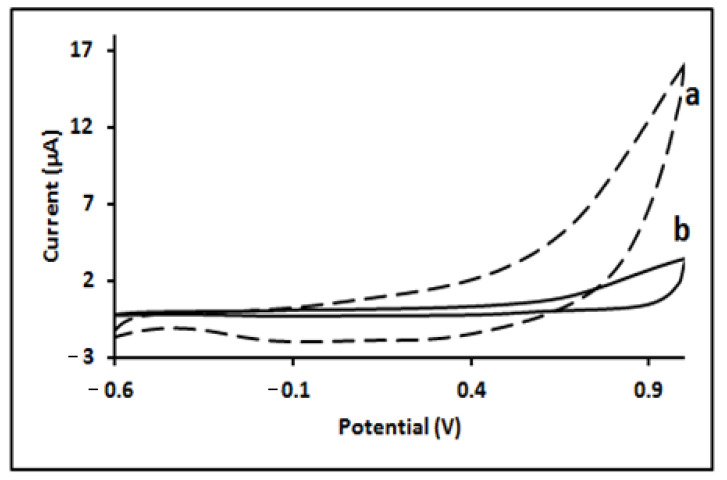
Cyclic voltammograms obtained during the preparation of (a) Non-imprinted polymer; and (b) STN-imprinted Polypyrrole at the GC electrode.

The molecularly imprinted polymer film was prepared by electro polymerisation on the surface of a bare GC electrode using CV in a potential range of −0.6 to 1.0 V and a scan rate 100 mV/S in B-R buffer solution (pH = 7) [39]. Figure 3 shows typical cyclic voltammograms recorded during the synthesis of MIP and NIP films. During the electro-polymerization of Py in the absence of a template, the oxidation of Py starts at 0.85 V in the first cycle and the oxidation peak intensity increased progressively on subsequent cycles, indicating the polymeric film growth on the working electrode [40]. Compared with NIP, the oxidation of Py in the presence of STN was delayed during the first cycle with a smaller peak intensity, remaining barely constant across consecutive cycles.

Reversible interactions between the STN molecule and the insoluble Py polymer network were expected to be formed since the process can be reversed and the sensor reused. The oxygen and/or nitrogen in the STN molecules would interact with hydroxyl groups in the polymer through hydrogen bonds and other possible non-covalent inter-molecular interactions. This complex chemistry would define the size and orientation of the chemical functions of the imprinted cavity.

#### 2.3.3. Optimization of the Imprinted Sensor

In the preparation of the imprinted sensor, some factors play an important role involving the concentrations of both functional monomers and templates and number of scanning cycles. At first, the number of scanning cycles was optimized. The number of scanning cycles during the electro polymerization can control the thickness of the polymer film. Generally, a thicker film is beneficial, since more imprinted sites can be achieved, but in case of a film being too thick, the template molecule might not be completely removed from the polymer [37] and conductivity may be reduced.

**Figure 4:**
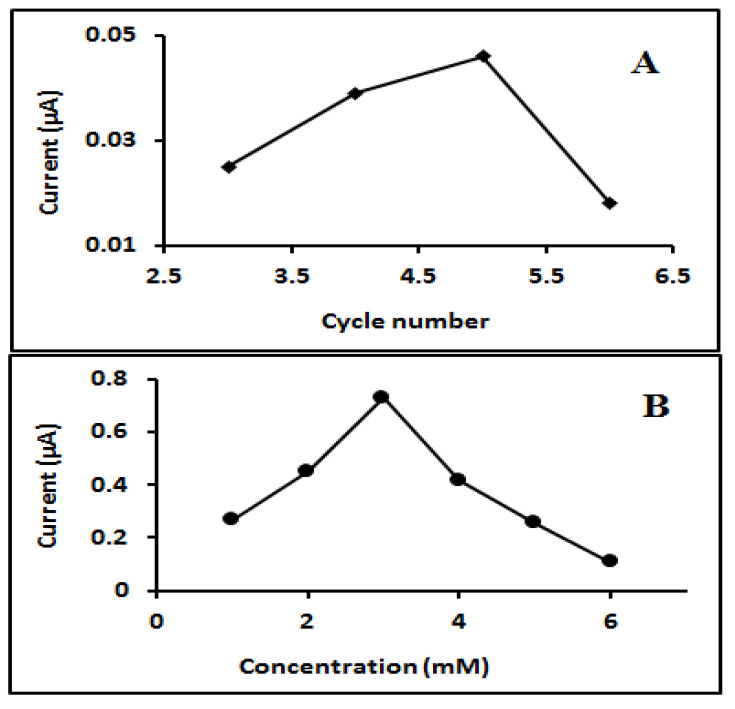
(**A**) Current response related to number of scan cycles used during electro polymerization of STN-Py at GC electrode; (**B**) current response related to increasing concentration of STN in electro polymerization solution of 5 mM Py.

In this study, different scanning cycles, including 1 cycle, 2 cycles, 3 cycles, 4 cycles and 5 cycles, were studied (Figure 4A). It was found that 3 cycles produced the best current response for the imprinted STN when compared to the NIP. The next variable to optimise was the template-monomer ratio, as it has a direct effect on the number of template molecules imprinted in the polymer film. The concentration of monomers should be higher than the template concentration and an excess concentration may affect the sensitivity of the formed MIP [37,41]. Various concentrations of STN (template) and a monomer concentration of 5 mM Py solution were evaluated for optimisation. When the proportion of STN concentration was increased in the polymer matrix, more STN molecules could be imprinted and be deposited in the polymer, producing better signal intensity on DPV. However, a greater concentration of STN interfered with the imprinted molecules and in turn affected the signal intensity. Therefore, optimal concentrations of 5 mM and 3 mM were chosen for PPY and STN, respectively (Figure 4B).

Extraction time for the template removal was a significant step in the preparation of molecularly imprinted electrochemical sensors. An acetic acid–acetonitrile (1:5, *v*/*v*) solution was used to elute the template molecules from the polymer. The imprinted polymer was incubated overnight in an extraction solution to allow complete removal of imprinted STN molecules.

#### 2.3.4. STN-MIP Sensor Voltammetry

**Figure 5:**
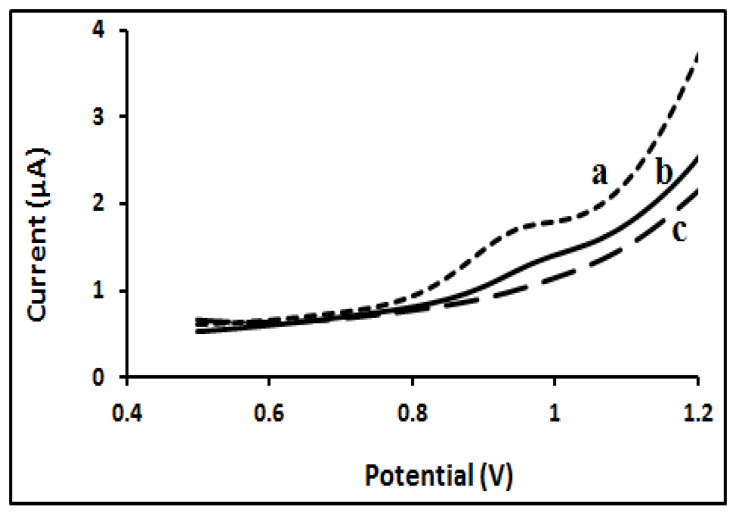
Differential pulse voltammetry of 50 µM STN in 0.1M BR buffer solution pH = 7, (a) on formed STN-MIP sensor; (b) on NIP electrode and (c) blank.

The developed sensor was studied by DPV under same conditions seen in Figure 4. In NIP and MIP, the polymeric film showed no oxidation peak over the potential range. A clear STN oxidation peak appeared when the STN was in solution in the presence of the STN-MIP sensor. The NIP response was close to that of the blank (Figure 5).

Furthermore, the potential peak of STN was slightly shifted to negative potentials compared to the results obtained in a bare GC electrode due to the formation of a new surface (MIP).

The voltammetric response of the developed MIP sensor was studied in BR buffer (pH 7). The peak current response was increased with the increasing the concentration of STN in the solution monitored by DPV. The peak currents were proportional to the concentration of STN in the range of 15–100 µM. The regression equation (Figure S18) was I (µA) = 0.0032 [STN (µM)] + 0.032 with r^2^ = 0.9907. The calculated limit of detection (LOD) and limit of quantitation (LOQ) for STN-MIP were 4.32 µM and 14.1 µM, respectively. The limit of detection is the smallest concentration of analyte that can be reliably distinguished from zero, whilst the limit of quantitation is the lowest amount of analyte which can be quantitatively determined with suitable precision and accuracy.

#### 2.3.5. Precision and Recovery

The precision of STN anodic peak at the GC electrode was estimated by calculating the percentage relative standard deviation (%RSD) for 5 repeated measurements on the same day (intra-day precision) and determination of 5 consecutive days (inter-day precision). The precision was assessed using triplicate measurements of three different concentrations of STN by DPV.

The calculated values for intra-day precision were of 2.36%, 4.95% and 2.32% for 25 µM, 50 µM, and 85 µM, respectively. Inter-day precision values were of 8.35%, 13.31% and 11.5% for 25 µM, 50 µM, and 85 µM, respectively. Overall, the average precision for all concentrations was less than 13.31%, indicating that the developed sensor is reliable and sufficiently precise. The recovery rate was calculated using three concentrations of STN prepared from the standard stock solution and three triplicates for each concentration by DPV. The recovery percentage for STN solutions with concentration of 25 µM, 50 µM, and 85 µM were 105.62%, 90.15%, and 92.03, respectively. The precision of these determinations (%RSD) were of 10.92%, 3.93%, and 2.49%, respectively. The combined recovery rate for all concentrations was larger than 90.15%, indicating that the developed sensor is fairly accurate for real applications.

#### 2.3.6. STN-MIP Sensor Selectivity

The selectivity of the developed STN-MIP sensor towards STN anodic peak in the presence of three potential interferents in the electrochemical determination was examined. Interferent compounds: Brucine, Scopolamine, and SNO were used for this assessment as these substances have oxidation peaks similar to that of STN and they are also structurally related. The current intensity in the STN oxidation peak in the presence of three individual different concentrations of interferents was recorded. Table 1 shows the comparison in the current response for a 55 µM STN solution in the presence of the same concentration for each interferent substances, utilising DPV measurements. In all cases, the signal change for anodic peak was less than 18.4%, indicating that the binding site in the imprinted sensor selectively recognises STN molecules, even in presence of potential interferents. Figure S19 shows the DPV figures for the compounds analysed in Table 1.

#### 2.3.7. Application of Developed Electrochemical Methods in Biological Matrix Media

To evaluate the validity of the proposed electrochemical method for the determination of STN and its major metabolites (SNO) in real-world biological samples, human plasma and human urine samples were spiked with known amounts of STN and SNO. One-mL of synthetic human plasma was added to 29 mL of BR buffer solution and 2 mL of fresh urine sample was also taken and diluted to 30 mL with the B-R buffer solution and was then directly analyzed. A-25 mM STN solution (Table 2) and 2 different concentrations of SNO, 100 mM and 200 mM, (Table 3) were spiked into the plasma and urine samples before CV or DPV analysis using the bare GC electrode or the STN-MIP sensor. Recoveries were similar for all cases, with a slightly improved performance for the STN-MIP, as it appeared to be less affected by the matrix effect with similar recoveries in both cases.

Table 3 includes the results obtained for DPV analysis of SNO using a GC electrode in urine and plasma samples. Generally, results were good, with a minimum percentage of recovery greater than 88.52%. Moreover, these results showed that the matrix did not significantly influence the recovered concentration, with urine having a slightly lesser effect on percentage recovery than plasma.

## 3. Materials and Methods

### 3.1. Chemical and Reagents

Strychnine (purity > 98%), Strychnine N-oxide (purity ≤ 100%) were obtained from Sigma (Sigma-Aldrich, Gillingham, UK). Phosphoric acid, Hydrochloric Acid, Glacial acetic acid and Potassium Hydroxide were obtained from Fisher Scientific (Loughborough, UK); and Sodium Chloride from Sigma Aldrich, UK. Britton-Robinson buffer solution (phosphoric acid, Glacial Acetic acid and Sodium Chloride) had the pH adjusted using sodium hydroxide and hydrochloric acid. Potassium ferricyanide from Sigma-Aldrich (St.Louis, MO, USA) was used to for the cyclic voltammetry testing of the polished glassy carbon electrode. Fumed silica (particle size 0.007 μm) and aluminium oxide (particle size 0.05 μm), used for polishing the glassy carbon electrode, were obtained from Sigma-Aldrich. Acetonitrile (HPLC grade) from Fisher Scientific, (Loughborough, UK); used in the sonication of the GC electrode. Water was purified using an ELGA purification system to a specific resistance 18 MΩ and used to prepare all solutions. Artificial human plasma was purchased from sigma (Sigma Aldrich, UK). The urine sample (ethics permission obtained) used was freshly supplied by a volunteer.

Pyrrole (Py) was obtained from Sigma (Sigma-Aldrich, UK). Brucine and scopolamine used in selectivity tests were bought from Sigma (Sigma-Aldrich, UK).

### 3.2. Instruments and Apparatus

Voltammetric experiments were performed using a Metrohm 757 VA Computrace (Metrohm Ltd., Runcorn, UK), the data processing software was Metrohm version 1.0 Ct757 software (Metrohm Ltd., Runcorn, UK), run using a personal computer (Compaq^®^ DeskPro, Windows^®^ 95). A conventional three electrode system was employed for all experiments, which consisted of a Glassy Carbon (GC) electrode as the working electrode (geometric working area of 0.0706 cm^2^), a Ag/AgCl electrode serving as a reference electrode (filled 3M KCl), and platinum as an auxiliary electrode. All electrodes were purchased from Metrohm Ltd. (UK). A digital pH meter (Hanna instrument microprocessor pH 210 m1ter) was used when preparing buffer solutions. An ultrasound bath (Kerry, London, UK) was used for electrode sonication. An Electronic balance was from Sartorius (Goettingen, Germany).

High Pressure Liquid Chromatography (Merck Hitachi, Tokyo, Japan) was employed in this study consisting of a L-7000 pump, a DAD-L-7455 diode array detector and an automated sampler L-7200. The column was an ultimate LP-C18 (250 × 4.6 mm^2^ I.D., 5 mm).

### 3.3. Solution Preparation

A stock solution of STN was prepared by dissolving STN in methanol to yield a concentration of 20 mM from which all other solutions were prepared. A stock solution of SNO (10 mM) was prepared by dissolving SNO in Methanol. All solutions were stored at −8 °C in amber bottles. All other working solutions were freshly prepared from standard stock solutions. One litre of 0.5 M Britton-Robinson buffer (B-R) was prepared, adding 33.8 mL of concentrated phosphoric acid (14.8 M), 28.6 mL of concentrated acetic acid (17.48 M) and 29.22 g of sodium chloride into one litre of distilled water. The pH value was adjusted with sodium hydroxide and hydrochloric acid.

### 3.4. Experimental Procedures

Prior to running all experiments, a glassy carbon electrode (GC) was polished to a mirror-like surface successively with activated aluminium oxide and 0.007 μ silica slurry. The electrode was thoroughly washed with deionised water and then treated with acetonitrile in an ultrasonic bath for about 5 min. Electrochemical experiments were carried out in a 50-mL voltametric cell at room temperature and electrochemical measurements were performed after initial purging of the mixture under nitrogen gas for 300 s. The cleaned bare GC electrode was tested by CV in 0.01 M K_3_[Fe(CN)_6_] solution with a scan rate of 0.1 V s^−1^ within the potential range of −1.0 to +1.0 V (vs. Ag/AgCl) until a pair of well-defined redox peaks were recorded.

Cyclic voltammetry (CV) measurements were achieved in the potential range from 0.7 to 1.2 V for strychnine and 0.3 to 1.0 V for strychnine N-oxide (SNO) with a scan rate of 100 mV s^−1^, and the equilibrium time is 10 s. Differential pulse voltammetry (DPV) measurements were performed in the potential range from 0.7 to 1.2 V for STN and 0.3 to 1.0 V for SNO with voltage step, 9.918 mV; pulse amplitude, 50 mV; pulse time, 0.04 s; voltage step time, 0.4 s, and sweep rate, 0.0248 V s^−1^.

One-mL of synthetic human plasma (Sigma Aldrich) was added to 29 mL of B-R buffer (pH 7) solution and 2 mL of fresh urine sample was diluted to 30 mL with the BR buffer solution; (without any pre-treatment) to prepare appropriate sample solutions and cyclic voltammogram and differential pulse voltammograms were as recorded under optimized conditions. Two different concentrations of STN (15 μM and 25 μM) and SNO (100 μM and 200 μM) were added into the plasma and urine samples before measuring with CV or DPV at the bare GC electrode.

HPLC under isocratic elution was conducted with a mobile phase consisting of a 12% buffer solution containing 30 mM ammonium acetate and 1% formic acid and 88% methanol (Sigma Aldrich). The monitoring wavelength was 253 nm. The oven temperature was set at 40 °C, and the flow rate was set to 1.0 mL min^−1^. For all the experiments, 20 μL of sample extract was injected.

### 3.5. Fabrication of GC Electrode with Imprinted Polymer

The electro polymerization was performed in an electrolyte solution which contained 8 mM pyrrole, 2 mM STN, and a 100 mM BR buffer (pH, 7) solution. Prior to electro-polymerization, the GC electrode was polished and then sonicated in methanol for 2 min.

The polymerization was performed by cyclic voltammetry in a potential range of −0.6 V to +1.0 V (vs. Ag/AgCl) with a scan rate of 0.1 V s^−1^ for 5 scan cycles, after initial purging of the mixture under nitrogen gas for 300 s. The STN molecules were removed from the polymeric film by immersing the MIP electrode into a stirred mixture of acetic acid and acetonitrile at a ratio of 1:5 (*v*/*v*). Finally, the molecularly imprinted GC electrode was then dried by blowing under nitrogen gas. The non-imprinted polymer (NIP) was prepared by following the same electro-polymerization and template removal steps but without the presence of the template molecule, STN, in the solution mixture of the electro-polymerisation step.

## 4. Conclusions

STN and its major metabolite (SNO) were examined electrochemically to facilitate the development of a reliable voltammetric method for the determination of STN and SNO in biological samples. Both the parent compound and its major metabolites showed electroactivity on glassy carbon electrodes using CV and DPV techniques. One oxidation peak at 1008 mV was observed for STN with the optimum peak intensity at pH 7. Two successive oxidation peaks at 617 mV and 797 mV were recorded for SNO at an optimum pH 5. The peaks demonstrated an irreversible behaviour and the irreversibility of the system was confirmed to different scan rates. A calibration curve was produced for both CV and DPV measurements and the sensitivity of the proposed EC method was good compared with previous electrochemical and non-electrochemical methods. The precision of oxidation peak of STN using the STN-MIP method agreed with the International Council for Harmonisation (ICH) guidelines, with a maximum value of 11.5% and 2.32% for inter-day and intra-day%RSD, respectively. The recovery percentage for STN solutions with concentrations of 25 µM, 50 µM, and 85 µM were 105.62%, 90.15%, and 92.035%, respectively. The electrochemical method has been successfully applied to the determination of STN in spiked plasma and urine samples with acceptable recovery rates, where the precision, recovery and accuracy of the MIP-STN did not differ for the bare glassy carbon electrode (values in Supplementary Materials).

For SNO, this is the first time this method has been reported in the literature. Both anodic peaks of SNO demonstrated an irreversible behaviour. A different sweep rate was used for calculating the number of ‘transfer electrons’ in the system; based on this, the mechanism of oxidation reaction was proposed. Calibration curves for both oxidative peaks were produced using DPV measurements. The second anodic peak demonstrated a high linearity and precision, with %RSD <1.96%. The obtained percentage of recovery showed good agreement compared to those reference values when HPLC was used as a reference method.

Both novel methods have been successfully applied for the quantification of SNO in spiked plasma and urine samples with an acceptable recovery rate. The suggested method would increase reliability and allow for fast measurements in vitro of strychnine and strychnine N-Oxide.

## Figures and Tables

**Table 1:** Effect of interferents on the differential pulse voltammetric response at the MIP electrode.

Interferent Molecules	Concentration (µM) ^a^	Signal Change (%) ^b^	RSD (%) (N = 3)
Brucine	67.08	18.4	1.77
SNO	45.36	14.96	7.01
Scopolamine	45.98	14.0	11.35

^a^ Spiked concentration to 55 µM STN solution. ^b^ Percentual increase of analytical signal following the addition of an interferent molecule.

**Table 2:** Recoveries from spiked urine and plasma samples for known concentrations of STN using CV and DPV measurements at a bare glassy carbon electrode and the STN-MIP.

Interference Media	Analytical Technique	Concentration Spiked (µM)	Mean (µM)	Recovered Percentage (%)
Plasma	GC/CV	25	20.2	80.8
	GC/DPV	25	20.7	82.8
	DPV/STN-MIP	25	21.9	87.6
Urine	GC/CV	25	22.1	88.4
	GC/DPV	25	25.7	102.8
	DPV/STN-MIP	25	21.7	86.8

**Table 3:** Recoveries from spiked urine and plasma samples for known concentrations of SNO using DPV measurements at bare glassy carbon electrode.

Interference Media	Concentration Spiked (µM)	Mean (µM)	Recovered Percentage (%)
Plasma	100	89.01	89.01%
	200	177.04	88.52%
Urine	100	93.6	93.6%
	200	183.2	91.6%

## Data Availability

The data that support the findings of this study are available from the corresponding author upon request.

## Supplementary Materials

The following are available online at https://www.mdpi.com/article/10.3390/molecules27061826/s1. Figure S1: (A) Cyclic voltammogram; (B) Differential pulse voltammogram; of (a) 1 mM STN and (b) blank solution, in 0.1 M Britton Robinson buffer solution (pH 7) on bare glassy carbon electrode at potential scan rate:100 mV. Figure S2: Differential Pulse voltammogram of 0.1 mM STN at pH value range (3–10) in 0.1 M BR buffer at bare GC electrode. Figure S3: (A) influence of pH on Potential peak; (B) influence of pH on current response; of 0.1 mM STN at pH value range (3–10) in 0.1 M BR buffer on bare GC electrode. Figure S4: Cyclic voltammogram of 1 mM STN in 0.1 M BR buffer solution (pH 7) on bare GC electrode at scan rates ranging 10–1000 mV/s. Figure S5: (A) The value of logarithm of intensity versus vs logarithm of scan rates ranging from 10–1000 mV/s for STN anodic peak. (B) Linear dependence of the peak potential of STN with the logarithm of scan rate ranging from 10–1000 mV/s. Figure S6: (A) The value of logarithm of Current intensity versus vs logarithm of scan rates ranging from 50–1000 mV/s; (B) Linear dependence of the peak potential with the logarithm of scan rate ranging from 50–1000 mV/s; for first oxidation peak of SNO. 100 µM of STN in 0.1 M BR buffer (pH 7) on bare GC electrode at scan rate of 100 mV/s. Figure S7: STN in 0.1M BR buffer (pH 7) on bare GC electrode. Figure S8: (A) Regression line for calibrated STN concentrations from CV measurements; (B) Regression line for calibrated STN concentrations from DPV measurements. Figure S9: Comparison of concentration values 15–100 µM STN obtain in the experimental set with GC/MS and CV (n = 3 for each concentration). Figure S10: Comparison of concentration values 15–100 µM STN obtain in the experimental set with GC/MS and DPV (n = 3 for each concentration). Figure S11: Differential Pulse voltammogram of 200 µM SNO at different pH values (3–8) in 0.1 M BR buffer at bare GC electrode. Figure S12: Influence of pH on potential peaks of SNO, (A) for second oxidation peak and (B) for first oxidation peak. Figure S13: Influence of pH on current response of, (A) second oxidation peak and (B) first oxidation peak; of 200 µM SNO in 0.1 M BR buffer on bare GC electrode. Figure S14: Cyclic voltammogram of 0.1 mM SNO in 0.1 M BR buffer solution (pH 5) on bare GC electrode at scan rates ranging (50–1000) mV/s. Figure S15: Cyclic voltammogram of 0.1 mM SNO in 0.1M BR buffer solution (pH 5) on bare GC electrode at scan rates ranging 50–1000 mV/s. (A) The value of logarithm of Current intensity versus vs logarithm of scan rates ranging from 50–1000mV/s; (B) Linear dependence of the peak potential with the logarithm of scan rate ranging from 50–1000 mV/s; for first oxidation peak of SNO. Figure S16: Differential pulse voltammogram for Seven concentrations of 25, 60, 100, 150, 200, 250, and 300 µM SNO in 0.1 M BR buffer (pH 5) on a bare GC electrode. Figure S17: (A) Regression line for calibrated SNO concentrations of first anodic peak measurements; (B) Regression line for calibrated SNO concentrations of second anodic peak measurements. Figure S18: Comparison for 15, 25, 40, 50, 55, 60, 70, 80, 85, 95 and 100 µM CFN obtained in the experimental set with GC/MS and DPV (n = 3 for each concentration). Figure S19: Regression line for calibrated STN concentrations at STN-MIP sensor using DPV measurements. Figure S20: DPV response from different spiked concentration to a 55 µM STN solution. From bottom to top: additions of 55 µM Scopolamine, 55 µM SNO and 55 µM Brucine. Table S1: Intra-day and inter-day precision for seven concentrations of STN using CV measurements. Table S2. Intra-day and inter-day precision for seven concentrations of STN using DPV measurements. Table S3: Recovery experiments for various concentrations of STN on bare GC electrode using CV measurements. Table S4: Recovery experiments for various concentrations of STN on bare GC electrode using DPV measurements. Table S5. Recovery experiments for various concentrations of SNO on bare GC electrode using DPV measurements.
