# Supplementary material for: The Production and Evaluation of an Electrochemical Sensors for Strychnine and Its Main Metabolite Strychnine N-Oxide for Their Use in Biological Samples
Source: Molecules. 2022 Mar 11;27(6):1826. doi: 10.3390/molecules27061826 (PMC8954432; doi:10.3390/molecules27061826)
Supplement: Supplementary file 1 [file molecules-27-01826-s001.zip › molecules-1621653-supplementary.pdf]

molecules

MDPI

Supplementary Materials

1. Strychnine

The anodic peak of STN was located at 1008 mV (vs. Ag/AgCl). - observed the oxidation peak of STN at potential of 1.17V using carbon fibre microcolumn electrode in 70 mM acetate buffer (pH 5.5) containing 0.01% (v/v) Brij35.

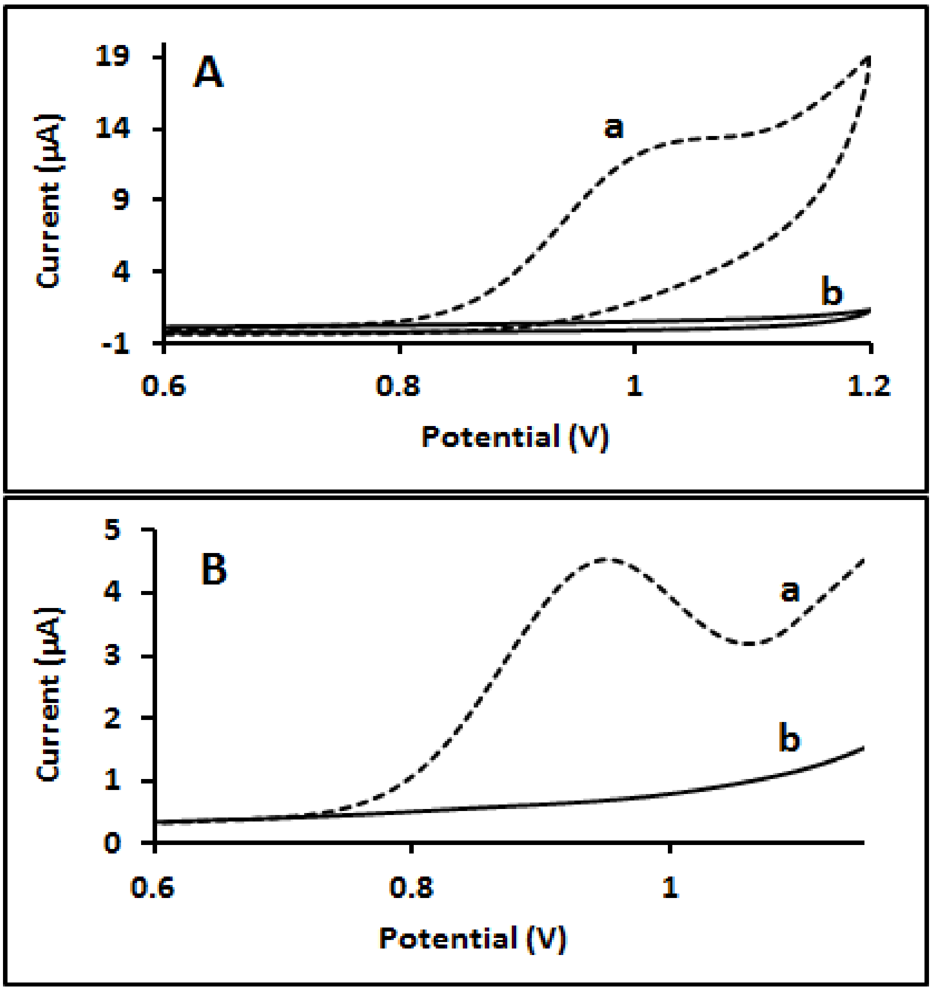

Figure S1. (**A**) Cyclic voltammogram; (**B**) Differential pulse voltammogram; of a) 1mM STN and b) blank solution, in 0.1 M Britton Robinson buffer solution (pH 7) on bare glassy carbon electrode at potential scan rate:100 mV.

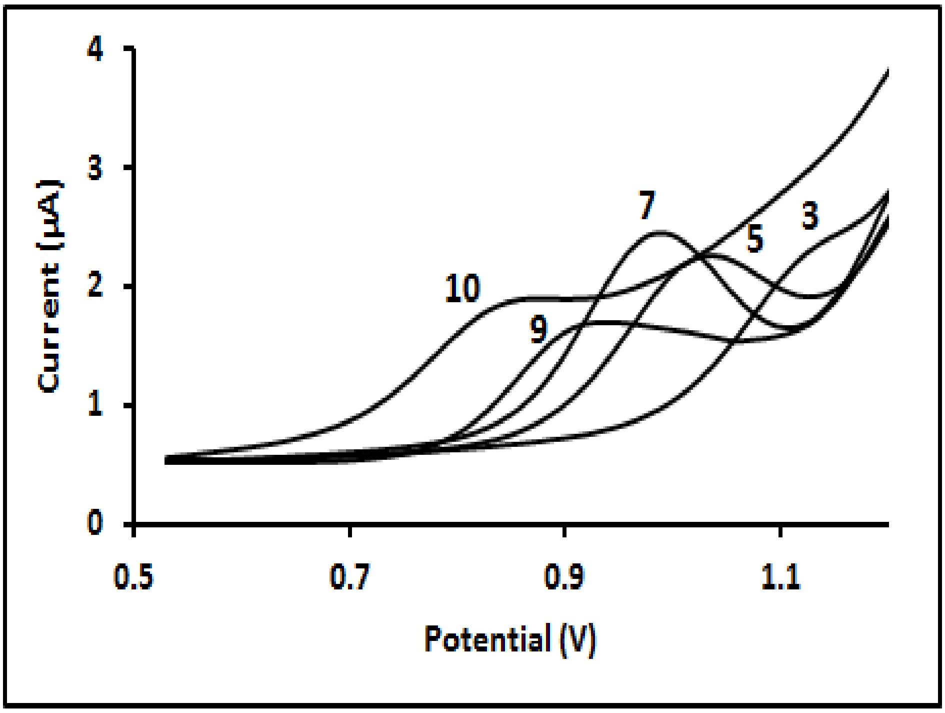

Figure S2. Differential Pulse voltammogram of 0.1mM STN at pH value range (3–10) in 0.1 M BR buffer at bare GC electrode.

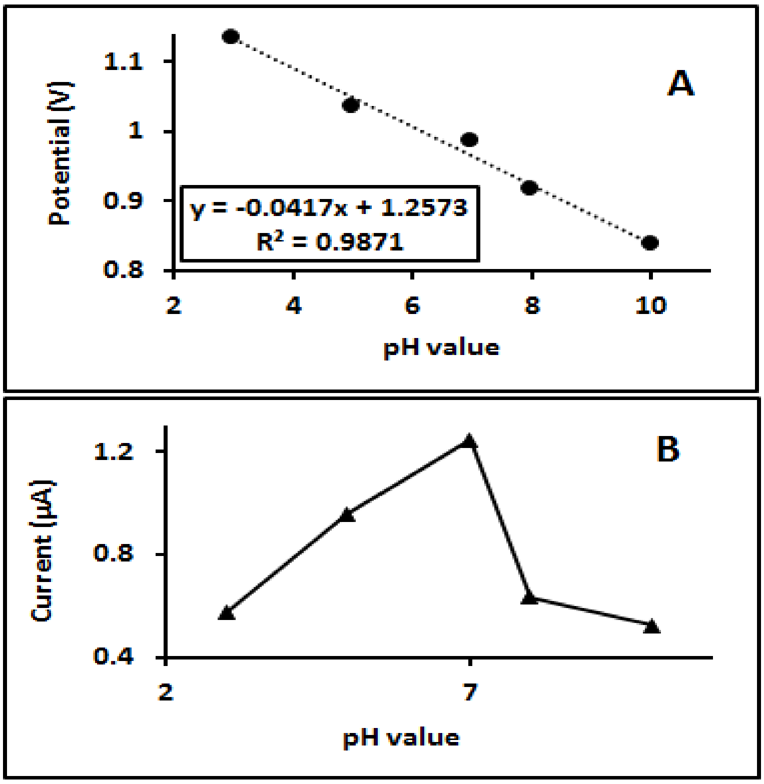

Figure S3. (**A**) influence of pH on Potential peak; (**B**) influence of pH on current response; of 0.1 mM STN at pH value range (3–10) in 0.1M BR buffer on bare GC electrode.

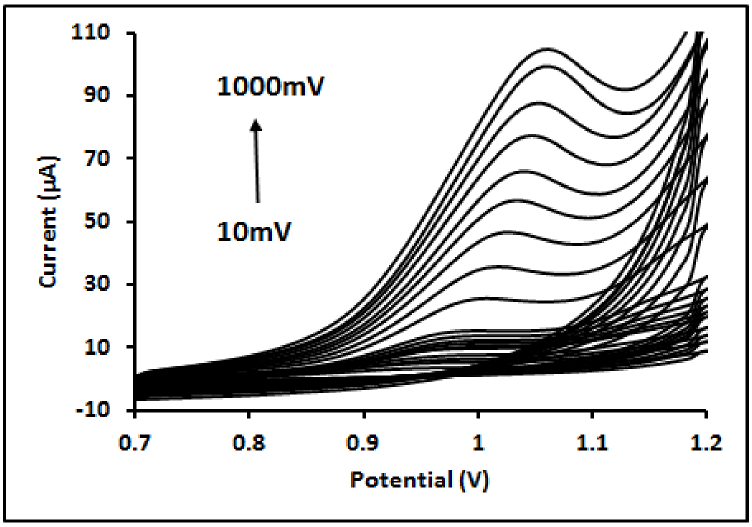

Figure S4. Cyclic voltammogram of 1 mM STN in 0.1 M BR buffer solution (pH, 7) on bare GC electrode at scan rates ranging (10–1000) mV/s.

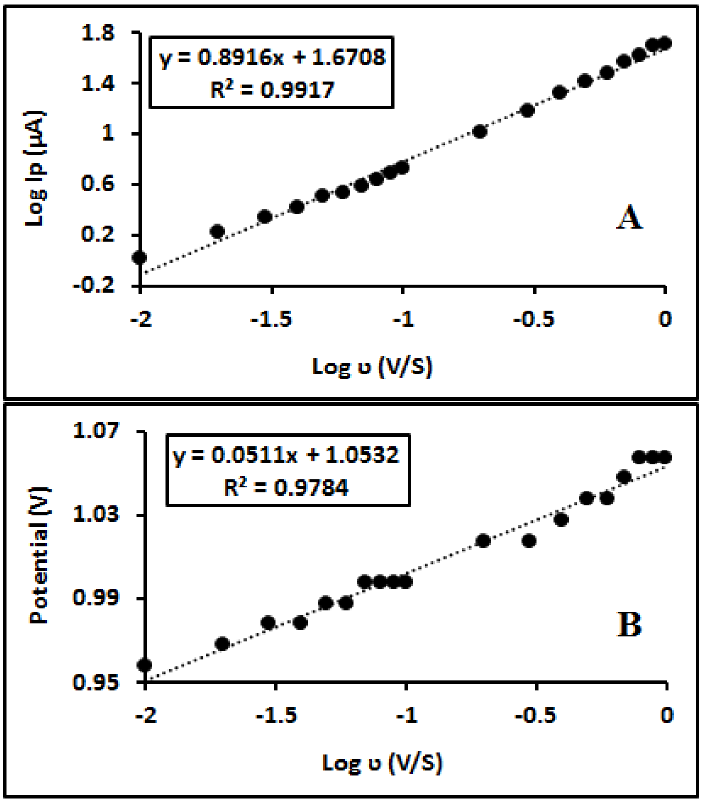

Figure S5. (**A**) The value of logarithm of intensity versus vs logarithm of scan rates ranging from 10-1000mV/s for STN anodic peak. (**B**) Linear dependence of the peak potential of STN with the logarithm of scan rate ranging from 10-1000mV/s.

According to Laviron's equation (1979) for irreversible species.

$$E_p = E^\circ + \left(\frac{2.303RT}{\alpha nF}\right)\log\left(\frac{RTK^\circ}{\alpha nF}\right) + \left(\frac{2.303RT}{\alpha nF}\right) \log \upsilon$$

the slope of Ep vs. log υ can be used for calculation of αn. Here the value of $\frac{2.303RT}{\alpha nF}$ is equal to 0.0511 and using the equation αn is equal to 1.15.

On other hand, based on Bard and Faulkner equation (2002), α can be calculated from this equation:

$$\alpha = 47.7/E_p\text{-}E_{p1/2}\ \text{mV}$$

where $E_{p/2}$ is the potential where the current is at half the peak value. For this system, we got the value of α to be 0.53.

Hence, the number of electrons (n) is shared in oxidation reaction of STN is equal to 2.14≈2. Hence, mechanism of oxidation of STN on the GC electrode surface is proposed as following in Figure 1 of the main paper.

2. Analytical study of the electrochemical behaviour of strychnine in a glassy carbon electrode.

2.1. Calibration curve

The STN peak showed a clear increment in the current intensity when concentrations were increased as showed in Figures S6,7. The calibration curves showed good linear responses within the concentration range from 10 to 100 μM and 5 to 100 μM using CV and DPV, respectively.

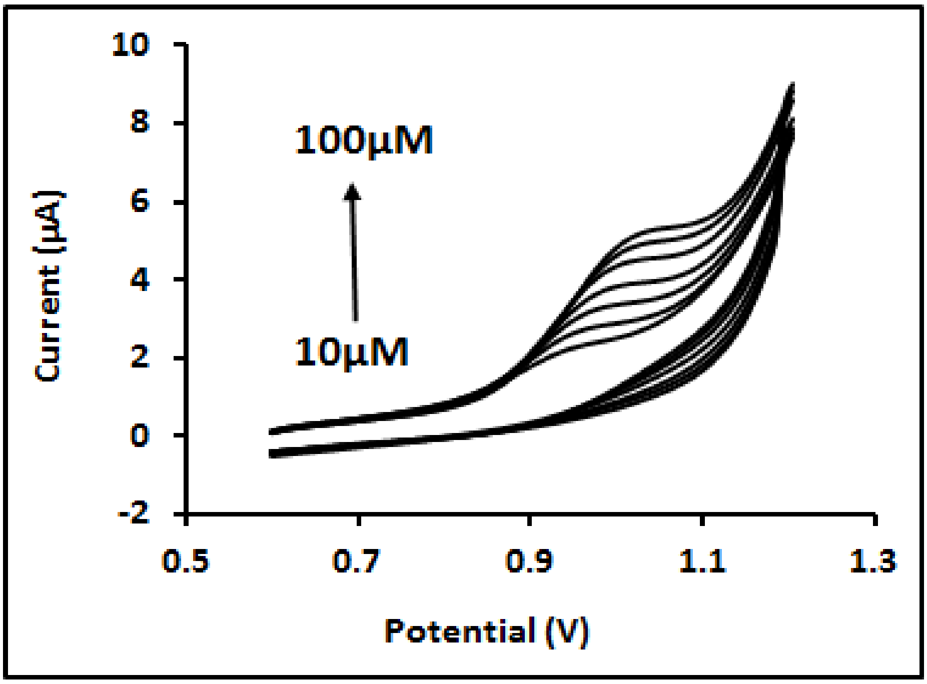

Figure S6. Cyclic voltammogram for Seven concentrations of (10, 25, 40, 55, 70, 85, and 100) μM of STN in 0.1 M BR buffer (pH 7) on bare GC electrode at scan rate of 100 mV/s.

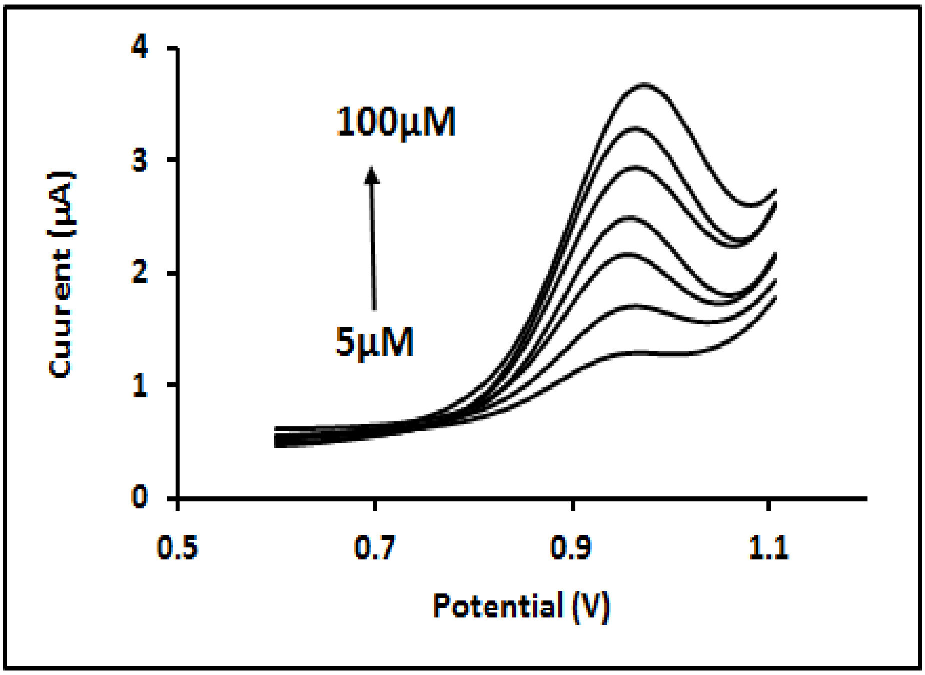

Figure S7. Differential pulse voltammogram for Seven concentrations of (5, 25, 40, 55, 70, 85, and 100) µM of STN in 0.1 M BR buffer (pH 7) on bare GC electrode.

The calibration graph for STN produced from the CV measurements was linear within the mentioned concentrations and fitted the equation I(A)=0.0184 [STN (µM)]+ 0.2275 with r^2=0.9983. While the calibration curve fitted the equation I(A)=0.0186 [STN (µM)]+0.1529 with r^2=0.9977 for STN analysed by DPV (Fig S8).

LOD was 1.02 µM and 0.71 µM, while LOQ was 3.1µM and 2.15µM for CV and DPV respectively. Thus, the sensitivity of the proposed method is nearly same compared to the electrochemical method used for the determination of STN in rat serum and in *Strychnos nux-vomica* seeds using carbon paste electrode modified with gold nanoparticles. Compared to non-electrochemical methods, the voltammetric method is also comparable with the GC-MS, CE, and HPLC methods suggested for the determination of various in biological samples. Clinically, the sensitivity of the developed method is enough to detect STN in blood sample after the appearance of toxicological signs.

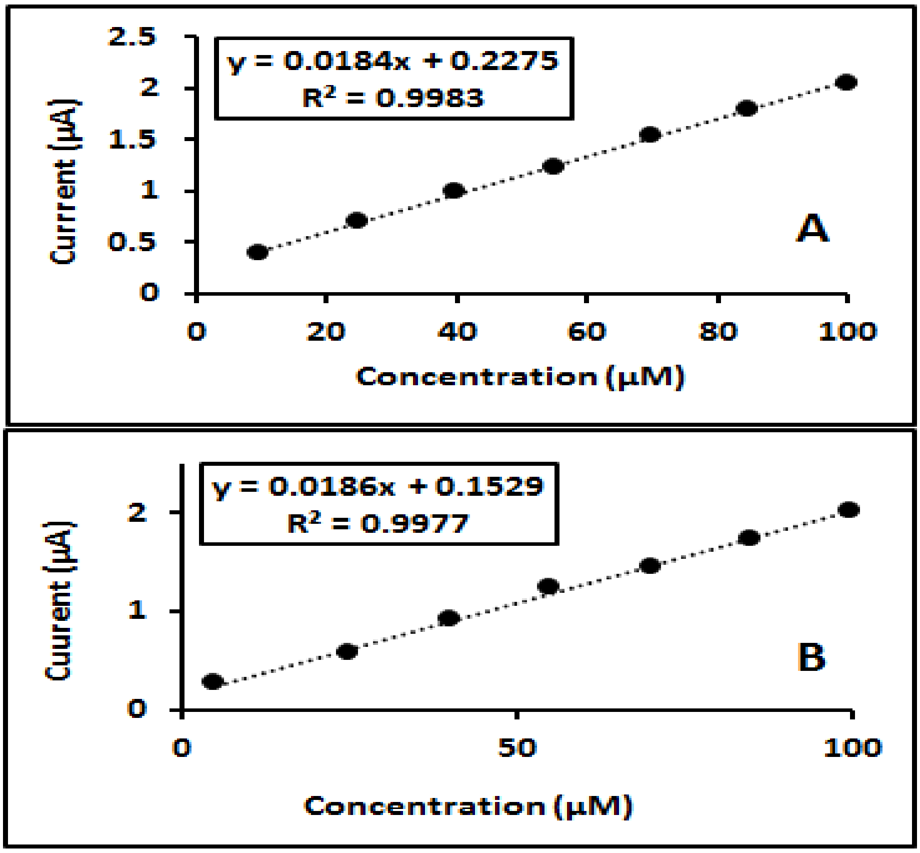

Figure S8. (**A**) Regression line for calibrated STN concentrations from CV measurements; (**B**) Regression line for calibrated STN concentrations from DPV measurements.

3. Precision

The precision of STN anodic peak at the GC electrode was estimated by calculating the percentage relative standard deviation (%RSD) for 5 repeated measurements on the same day (intra-day precision) and determination of 5 consecutive days (inter-day precision). The precision was assessed for seven concentrations of STN in both CV and DPV techniques shown in Table S1 and S2.

Table S1. Intra-day and inter-day precision for seven concentrations of STN using CV measurements.

Concentration (µM)	intra-day precision		Inter-day precision	
	Mean ±SD (µM)	RSD (%)	Mean ±SD (µM)	RDS (%)
10	6.6±0.91	13.87%	9.53±1.57	16.47%
25	25.02±2.03	8.14%	23.16±3.45	14.63%
40	40.4±4.01	10.1%	41.22±4.25	10.32%
55	54.37±3.02	5.56%	54.97±3.13	5.57%
70	71.44±3.32	4.64%	73.23±3.8	5.19%
85	82.41± 2.52	3.06%	83.45±2.89	3.46%
100	93.5±1.91	2.04%	95.95±3.54	3.69%

Table S2. Intra-day and inter-day precision for seven concentrations of STN using DPV measurements.

Concentration (µM)	Intra-day precision		Inter-day precision	
	Mean ±SD (µM)	RSD (%)	Mean ±SD (µM)	RDS (%)
5	6.08±0.39	6.49%	7.37±1.04	14.76%
25	23.01±2.63	11.45%	25.32±2.95	11.67%
40	46.72±4.69	10.05%	42.74±5.2	12.17%
55	60.32±1.96	3.25%	57.58±2.07	3.6%
70	73.71±3.21	4.36%	68.66±3.33	4.85%
85	84.46±2.66	3.16%	87.63±3.06	3.49%

100	100.08±1.75	1.73%	97.47±2.56	2.63%

In CV, precision was achieved in the range 2.04-13.87% and 3.46–16.47% for intra-day and inter-day measurements, respectively. In DPV, precision was achieved in a range 1.73-11.45% and 2.63-14.76% for intra-day and inter-day measurements, respectively. Overall, the precision for all concentrations and with both CV and DPV techniques is less than 16.47%, indicating that developed sensor is reliable and precise.

4. Recovery

The recovery was assessed for fifteen concentrations of STN prepared from standard stock solution employing both CV and DPV techniques shown in Table S3 and S4.

The percentage recovery obtained ranged from 92.76-104.02% and 92.15-104.96% using CV and DPV, respectively. Thus, the recovery rate in all concentrations and with both CV and DPV is larger than 92.15%, indicating that developed EC method is reliable for application. To validate the developed method, same concentrations of STN were also injected with GC-MS, used as a reference method, to compare with recovery results from electrochemical system. The recovered concentration was calculated from their calibration curve. The results depicted a straight linear relationship between the recovered concentrations (Figures S9,S10).

Table S3. Recovery experiments for various concentrations of STN on bare GC electrode using CV measurements.

Concentration (µM)	Recovered concentration (N=3)		
	Mean ±SD (µM)	RSD (%)	Recovered percentage (%)
10	8.37±1.3	15.51%	83.78%
15	12.27±1.98	16.2%	81.82%
25	22.3±3.3	15.01%	88.94%
30	31.74±1.32	3.85%	105.8%
35	34.46±4.99	14.48%	98.47%
40	39.9±4.4	11.04%	99.74%
50	49.04±7.11	14.51%	98.09%
55	53.03±0.92	1.74%	96.42%
60	58.65±4.7	8.02%	97.75%
70	72.41±2.21	3.06%	103.46%
80	80.93± 1.63	2.02%	101.16%
85	81.92±2.78	3.39%	96.38%
90	92.43±1.0	1.08%	102.4%
100	102.12±6.67	6.54%	102.12%

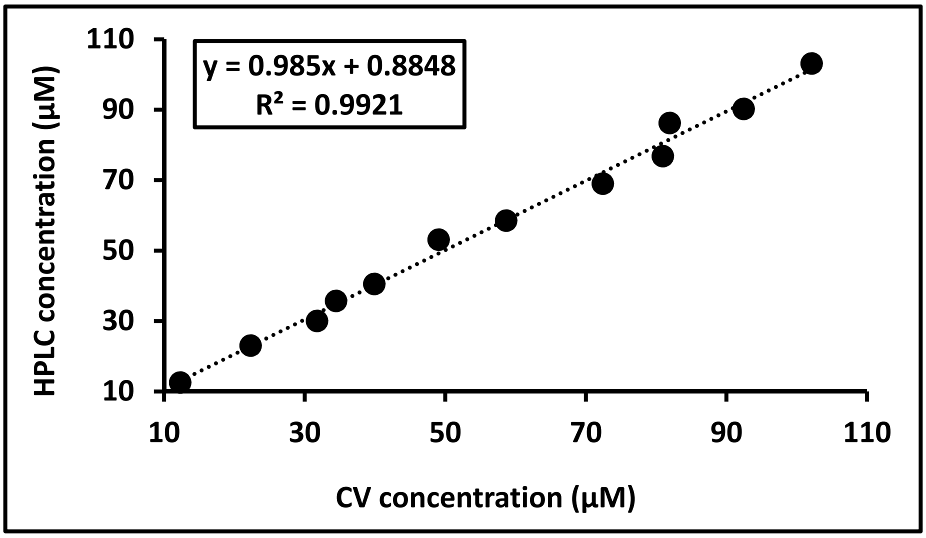

Figure S9. Comparison of concentration values (15 to 100) μM STN obtain in the experimental set with GC/MS and CV (n = 3 for each concentration).

Table S4. Recovery experiments for various concentrations of STN on bare GC electrode using DPV measurements.

Concentration (μM)	Recovered concentration (N=3)		
	Mean ±SD (μM)	RSD (%)	Recovered percentage (%)
5	5.13±0.63	12.34%	102.61%
15	14.71±1.12	7.65%	98.12%
25	20±1.53	7.68%	80.02%
30	27.62±1.64	5.96%	92.07%
35	33.17±2.01	6.06%	94.79%
40	42.31±3.04	7.18%	105.79%
50	48.85±1.18	2.43%	97.71%
55	56.56±4.4	7.79%	102.84%
60	62.29±4.72	7.57%	103.83%
70	66.24±4.85	7.32%	94.63%
80	78.09±2.95	3.78%	97.58%
85	80.22±4.18	5.22%	94.37%
90	87.03±5.93	6.81%	96.7%
100	98.05±4.95	5.05%	98.05%

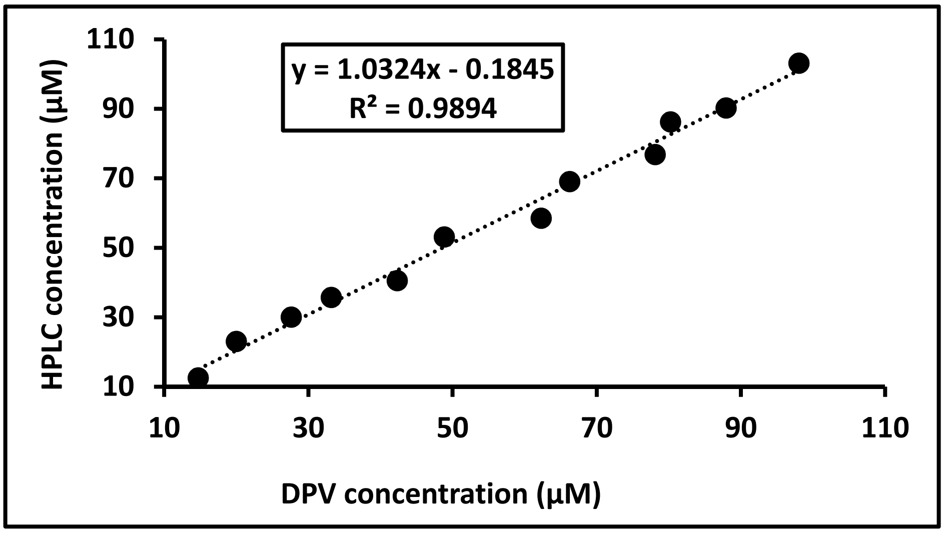

Figure S10. Comparison of concentration values (15 to 100) μM STN obtain in the experimental set with GC/MS and DPV (n = 3 for each concentration). .

5. Strychnine N-Oxide

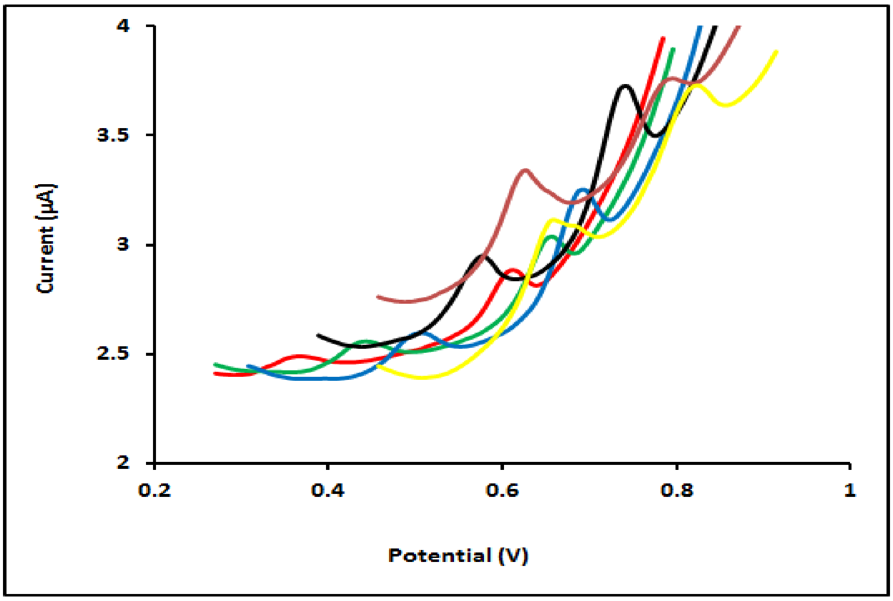

Figure S11. Differential Pulse voltammogram of 200µM SNO at different pH values (3–8) in 0.1 M BR buffer at bare GC electrode.

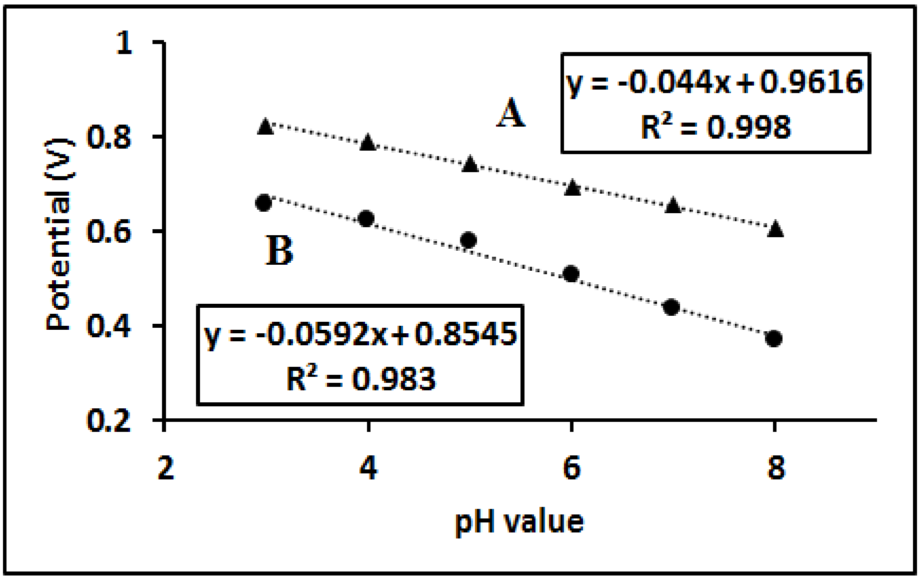

Figure S12. Influence of pH on potential peaks of SNO, (**A**) for second oxidation peak and (**B**) for first oxidation peak.

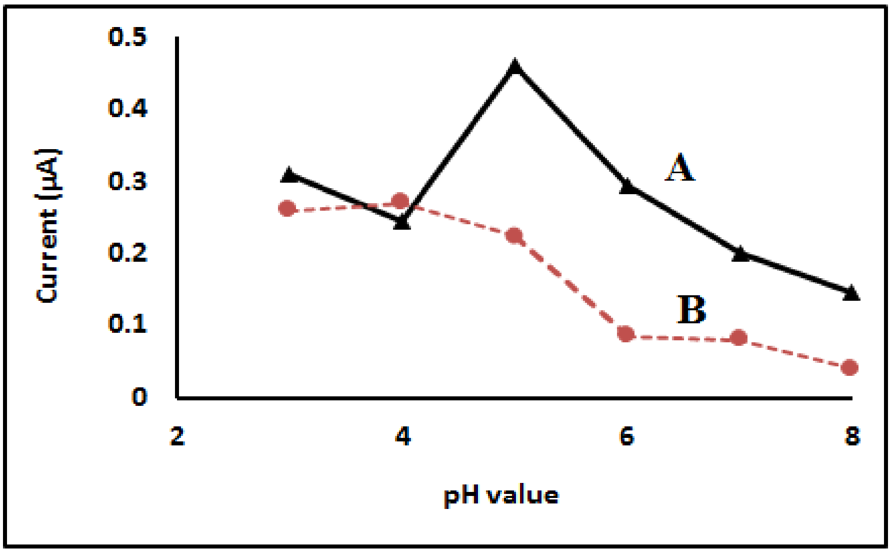

Figure S13. Influence of pH on current response of, (**A**) second oxidation peak and (**B**) first oxidation peak; of 200 μM SNO in 0.1M BR buffer on bare GC electrode.

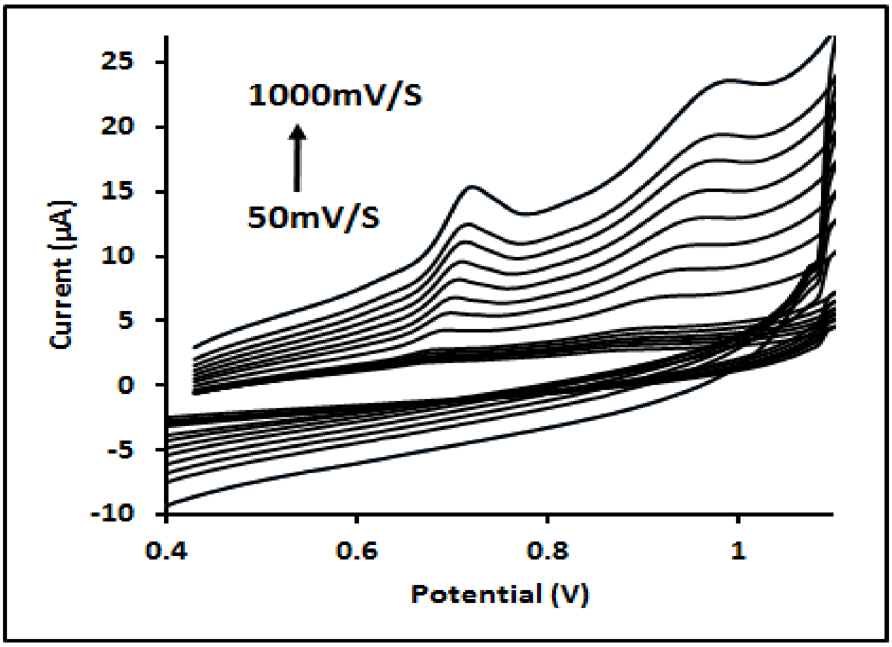

Figure S14. Cyclic voltammogram of 0.1 mM SNO in 0.1M BR buffer solution (pH, 5) on bare GC electrode at scan rates ranging (50–1000) mV/s.

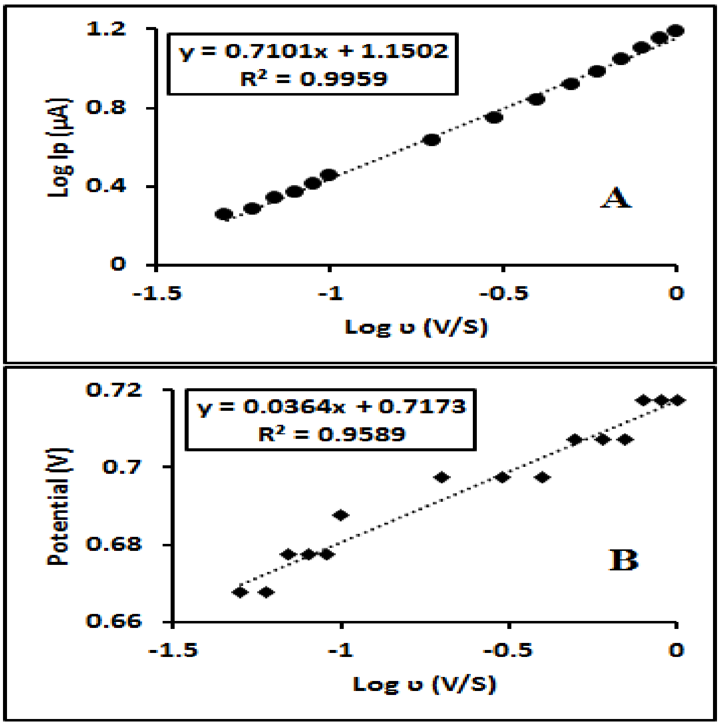

Figure S15. (A) The value of logarithm of Current intensity versus vs logarithm of scan rates ranging from 50–1000 mV/s; (B) Linear dependence of the peak potential with the logarithm of scan rate ranging from 50–1000 mV/s; for first oxidation peak of SNO.

A plot of peak potential (E_p) versus logarithm scan rate (log υ) was performed in the sweep rate range 50-1000mV/s to evaluate the number of transferred electrons in the first oxidation peak of SNO (Figure S14). The linear relationship was obtained as follow equation:

$$\text{Log } E_p=0.0364 \log \upsilon + 0.7173,\ R^2=0.9589$$

On other hand, according to Laviron's equation for irreversible species.

$$E_p= E^\circ + \left(\frac{2.303RT}{\alpha nF}\right)\log\left(\frac{RTK^\circ}{\alpha nF}\right) + \left(\frac{2.303RT}{\alpha nF}\right) log\, \upsilon$$

Accordingly, the slope of Ep vs. log υ can be used for calculation of αn. Here the value of $\frac{2.303RT}{\alpha nF}$ is equal to 0.0364 (Gowda and Nandibewoor, 2014) and from calculation, αn is equal to 1.61 from slope of equation (0.0482).

Based on Bard and Faulkner equation, α can be calculated from this equation:

$\alpha = 47.7/E_p - E_{p1/2}$ mV

where $E_{p/2}$ is the potential where the current is at half the peak value. So, from this we got the value of α to be 0.90. Further, the number of electrons (n) is shared in oxidation reaction of SNO is equal to 1.77≈2.

6. Calibration curve

The anodic peaks of SNO analysed by DPV were clearly proportional to the the concentration of the analyte in the supporting electrolyte solution, as showed in Figure S15. The calibration curves showed good linear responses within the concentration range from 25 to 300 µM with correlation coefficient r^2 = 0.9722 and 0.9945 for first and second anodic peaks, respectively (Fig.S17 A and B).

The LOD was 4.06 µM and 3.77 µM while LOQ was 12.3 µM and 11.42 µM for first and second peaks, respectively.

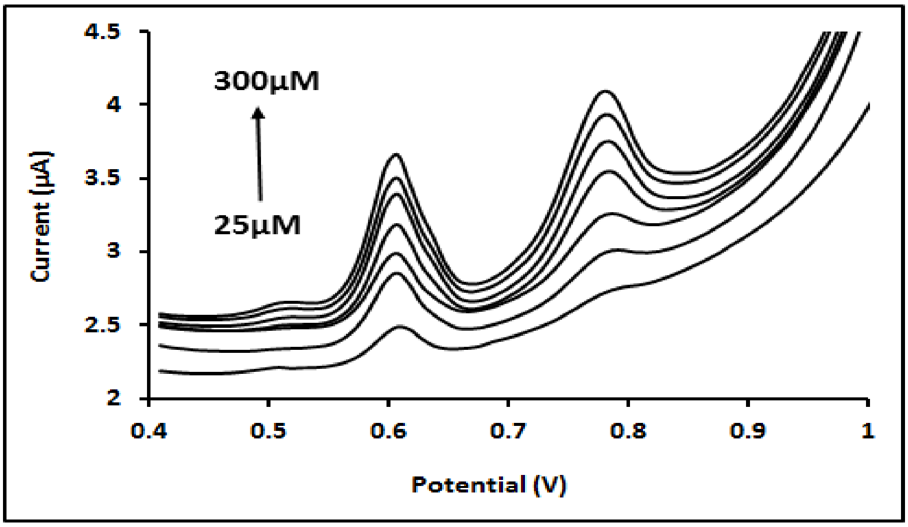

Figure S16. Differential pulse voltammogram for Seven concentrations of 25, 60, 100, 150, 200, 250, and 300 µM SNO in 0.1M BR buffer (pH,5) on a bare GC electrode.

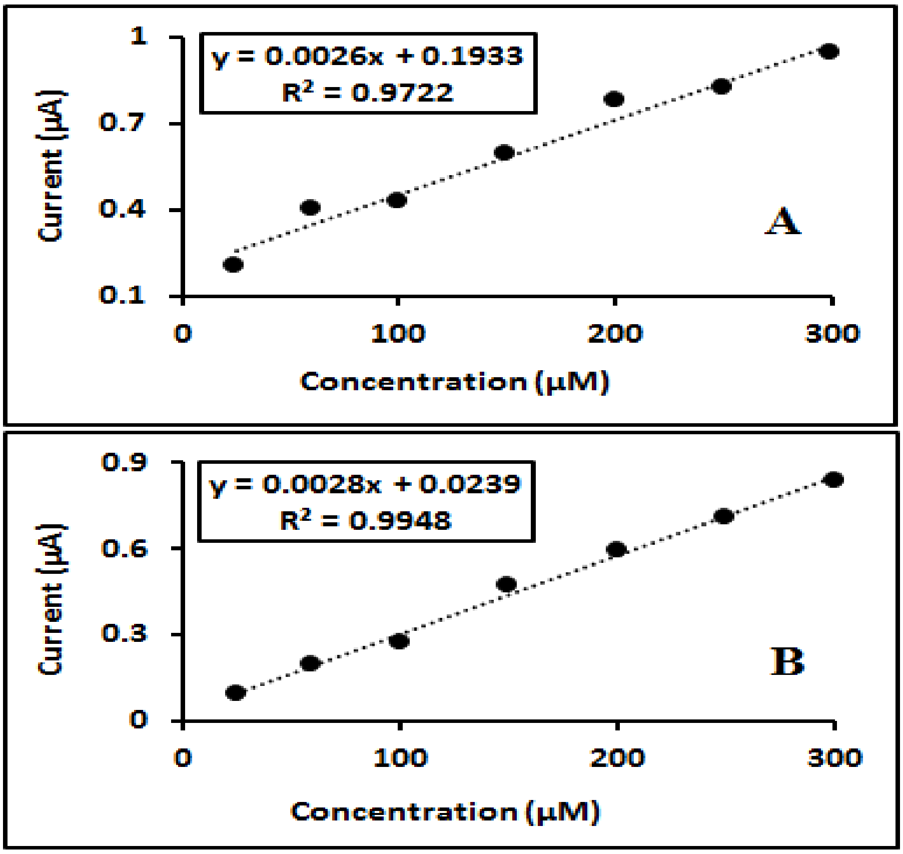

Figure S17. (**A**) Regression line for calibrated SNO concentrations of first anodic peak measurements; (**B**) Regression line for calibrated SNO concentrations of second anodic peak measurements.

7. Precision

The precision of SNO anodic peak at the GC electrode was estimated by calculating the percentage relative standard deviation (%RSD) for 5 repeated measurements on the same day (intra-day precision) and determination of 5 consecutive days (inter-day precision). The precision was assessed using triplicates of SNO by DPV. The intra-day precision values were 7.7, 4.33 and 0.6% and inter-day 16.96, 11.28 and 0.9% for 25µM, 150µM, and 300µM, respectively. Overall, the precision for all concentrations is less than 16.96%, indicating that developed sensor is reliable and precise.

8. Recovery

The recovery was assessed for fifteen concentrations of SNO prepared from the standard stock solution employing DPV as shown in Table 7.5. The recovery percentage obtained ranged from76.6–103.2%. Thus, the recovery rate in all concentrations is larger than 76.6%, indicating that developed EC method is reliable for application. To validate the developed method, same concentrations of SNO were also injected with GC-MS as a reference method to compare with recovery results from electrochemical system. The recovered concentration was calculated from their calibration curve. The results depicted a straight linear relationship between the recovered concentrations (Fig.S18).

Table S5. Recovery experiments for various concentrations of SNO on bare GC electrode using DPV measurements.

Concentration (μM)	Recovered concentration (N=3)		
	**Mean ±SD (μM)**	**RSD (%)**	**Recovered percentage (%)**
25	19.1±3.34	17.1%	76.6%
60	61.19±10.4	16.8%	103.2%
100	103.2±10.8	10.5%	103.2%
150	140±13.3	9.78%	93.3%
200	199.01±8.29	4.16%	99.6%
250	241.2±3.1	2.15%	96.48%
300	292.35±1.7	0.6%	97.45%

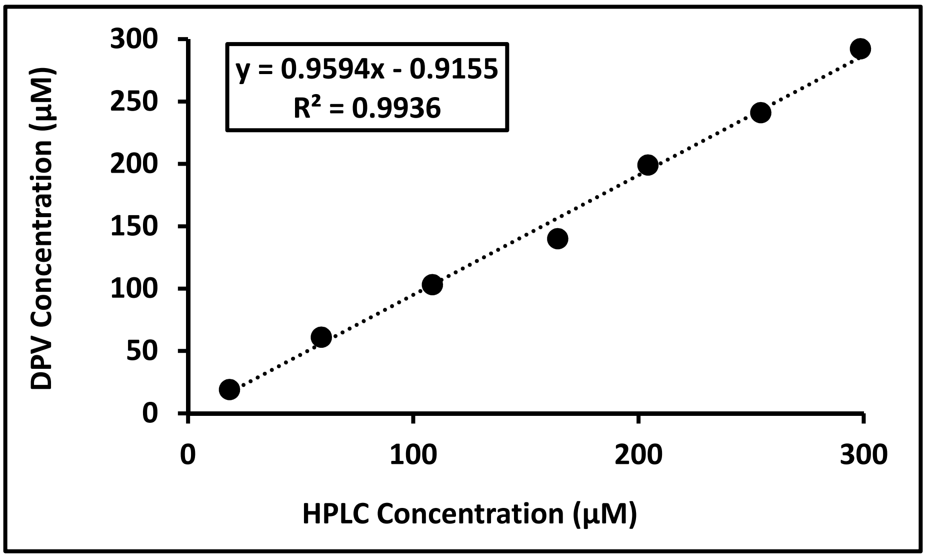

Figure S18. Comparison for 15, 25, 40, 50, 55, 60, 70, 80, 85, 95 and 100 μM CFN obtained in the experimental set with GC/MS and DPV (n = 3 for each concentration).

9. STN-MIP

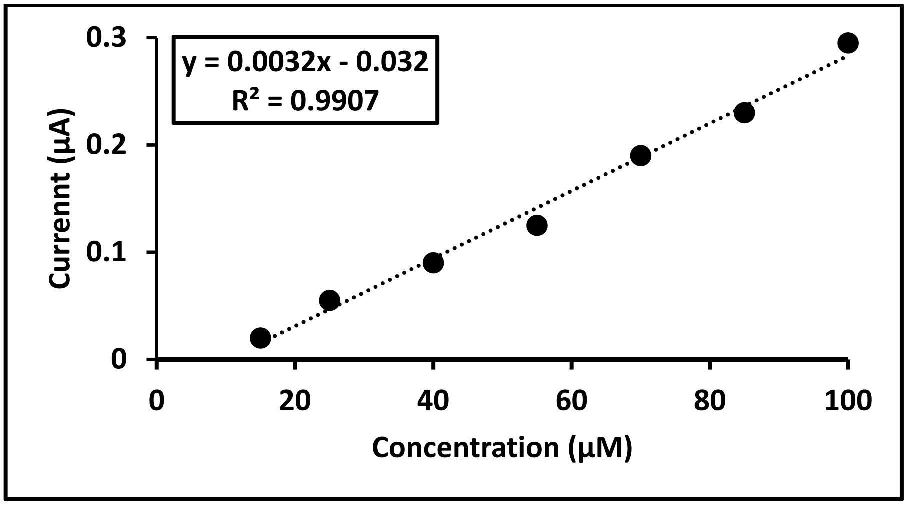

Figure S19. Regression line for calibrated STN concentrations at STN-MIP sensor using DPV measurements.

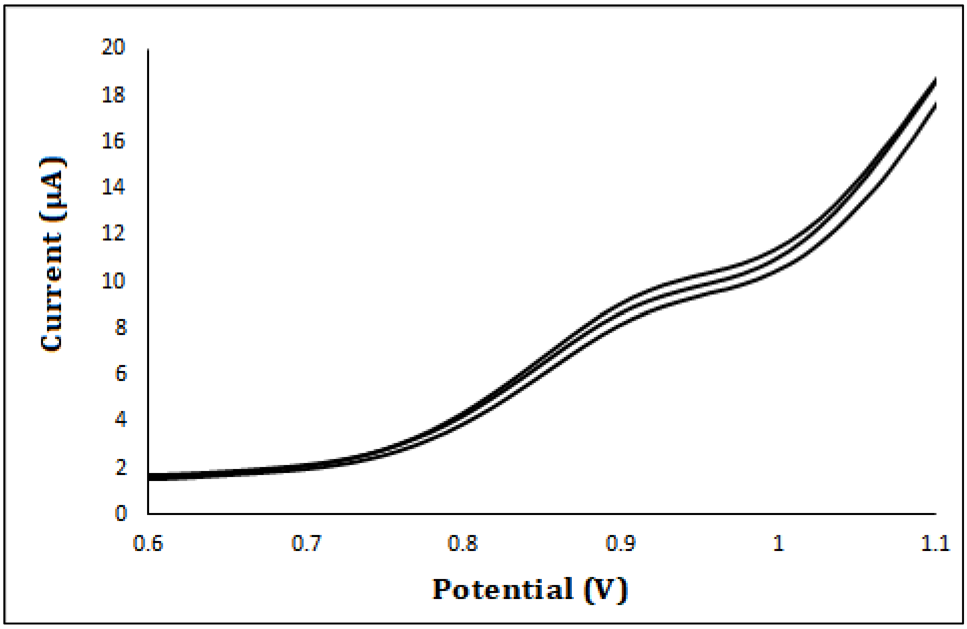

Figure S20. DPV response from different spiked concentration to a 55 µM STN solution. From bottom to top: additions of 55 µM Scopolamine, 55 µM SNO and 55 µM Brucine.
